# Green Synthesis of Smart Metal/Polymer Nanocomposite Particles and Their Tuneable Catalytic Activities

**DOI:** 10.3390/polym8040105

**Published:** 2016-03-23

**Authors:** Noel Peter Bengzon Tan, Cheng Hao Lee, Pei Li

**Affiliations:** Department of Applied Biology and Chemical Technology, The Hong Kong Polytechnic University, Hung Hom, Kowloon, Hong Kong, China; bengzontan@nami.org.hk (N.P.B.T.); chenghao.lee@polyu.edu.hk (C.H.L.)

**Keywords:** gold nanoparticles, silver/gold bimetallic nanoparticles, smart core-shell microgel, metal/polymer nanocomposite, reduction of *p*-nitrophenol

## Abstract

Herein we report a simple and green synthesis of smart Au and Ag@Au nanocomposite particles using poly(*N*-isopropylacrylamide)/polyethyleneimine (PNIPAm/PEI) core-shell microgels as dual reductant and templates in an aqueous system. The nanocomposite particles were synthesized through a spontaneous reduction of tetrachloroauric (III) acid to gold nanoparticles at room temperature, and *in situ* encapsulation and stabilization of the resultant gold nanoparticles (AuNPs) with amine-rich PEI shells. The preformed gold nanoparticles then acted as seed nanoparticles for further generation of Ag@Au bimetallic nanoparticles within the microgel templates at 60 °C. These nanocomposite particles were characterized by TEM, AFM, XPS, UV-vis spectroscopy, *zeta*-potential, and particle size analysis. The synergistic effects of the smart nanocomposite particles were studied via the reduction of *p*-nitrophenol to *p*-aminophenol. The catalytic performance of the bimetallic Ag@Au nanocomposite particles was 25-fold higher than that of the monometallic Au nanoparticles. Finally, the controllable catalytic activities of the Au@PNIPAm/PEI nanocomposite particles were demonstrated via tuning the solution pH and temperature.

## 1. Introduction

Metal nanoparticles have gained much attention over the past two decades because of their unique properties. Their distinctive size and shape-dependent optical and electronic properties have opened up vast potential applications in various fields [1]. However, metallic nanoparticles have a tendency to aggregate due to their large surface energy, which drives the thermodynamically favored coalescence process. Various types of stabilizing agents have been used to prevent nanoparticles from aggregating. They include ligands [2], surfactants [3,4], polymers [5,6], dendrimers [7], cyclodextrin [8], and microgels [9]. Recently, the use of smart polymer particles to stabilize metallic nanoparticles has received increasing attention because of the synergistic properties of the smart metal/polymer nanocomposite particles, derived from responsive polymer and metallic nanoparticles [10,11]. Smart metal/polymer nanocomposite particles of this kind have shown promising potential for use as hybrid materials for biomedical applications [12,13], sensing [14], and catalytic reactions [15,16].

Our group has recently reported a simple and green synthesis of AuNPs/polymer nanocomposite particles through a spontaneous reduction of tetrachloroauric (III) acid and encapsulation of the resultant gold nanoparticles using amine-rich core-shell particles in water [17,18]. The high local amine concentration of the polyethyleneimine shell enables effective reduction of gold ions to gold nanoparticles in the absence of any organic solvents, reducing agents, or stabilizers. Thus we envision that smart metal/polymer nanocomposite particles synthesized by this green approach may find potential application in catalytic reactions because the microgel particles can not only stabilize the metal nanoparticles and retain their nanoscale properties, but also allow the switching on and off of the catalytic activity of the metal nanoparticles through controlling the accessibility of the metal nanoparticles to the reactant. Moreover, the nanocomposite particles can be easily recovered and reused. The nanocomposite particles containing bimetallic nanoparticles are of particular interest because of their possible synergistic effects [19]. For example, Ahn and co-workers have recently reported the synthesis of Ag@Au bimetallic nanoparticles on magnetic silica microspheres through seeding, coalescing, seeds to cores, and then growing shells from the cores on aminopropyl functionalized silica microspheres [20]. Xin *et al.* have also synthesized Au@Ag bimetallic nanoparticles using polyelectrolyte multilayers (PEMs) of poly(styrene sulfonate) (PSS) and poly(diallyldimethylammonium chloride) (PDDA) particles as support [21]. Electrostatic interaction between the cations and anions of the PEMs and metal ions was used as a driving force to attract metal ions into the system. Subsequent reduction of metal salts with reducing agents such as NaBH_4_ and ascorbic acid was carried out in repeated cycles to improve the loading and size of the metal nanoparticles. However, current methods for generating bimetallic nanoparticles usually involve tedious procedures that are not amenable for scale-up production. In this study, we have synthesized a dual responsive microgel particle that consists of a temperature-sensitive core of poly(*N*-isopropylacryamide) and a pH-sensitive polyethyleneimine shell.. This type of pH- and temperature-responsive microgel particles was used to generate silver in gold Ag@Au/polymer nanocomposite particles. The synergistic effects of the smart nanocomposite particles were studied using the reduction of *p*-nitrophenol to *p*-aminophenol as a model reaction. The controllable catalytic activity of the Au@PNIPAm/PEI nanocomposite particles was demonstrated via the tuning of solution pH and temperature.

## 2. Materials and Methods

### 2.1. Materials

Spindle-crystals of *N*-isopropylacrylamide (NIPAM, Sigma-Aldrich, Saint Louis, MO, USA) were purified by repeated recrystallization in a mixture of toluene and n-hexane (1:5 *v*/*v*). Branched polyethyleneimine (PEI) with an average molecular weight of 750,000 (50 wt % solution in water), *N,N*-methylenebisacrylamide (MBA) and *tert-*butyl hydroperoxide (TBHP, 70% aqueous solution), hydrogen tetrachloroaurate (III) trihydrate (HAuCl_4_·3H_2_O), silver nitrate (AgNO_3_), *p*-nitrophenol (reagent grade), and sodium borohydride were all purchased from Sigma-Aldrich Chemical Co., and used as received. Deionized water or Milli-Q water was used for dilution and dispersion medium.

### 2.2. Synthesis of Au@PNIPAm/PEI Nanocomposite Particles

The synthesis of Au-loaded poly(*N*-isopropyl acrylamide)/polyethyleneimine (PNIPAm/PEI) nanocomposite particles was based on our previously established method [17]. Such a method involved a simple mixing of preformed PNIPAm/PEI microgel particles with a hydrogen tetrachloroaurate (III) trihydrate (HAuCl_4_·3H_2_O) solution according to the following general procedure: A stock solution of hydrogen tetrachloroaurate (III) trihydrate (1.317 × 10^−3^ M) was purged with N_2_ for 30 min. It was then added dropwise (1 mL) to the PNIPAm/PEI microgel dispersion (20 mL, 400 ppm, molar ratio of N/Au^3+^ = 28). The mixture was stirred at 250 rpm for 4 h at 25 °C. The resultant gold-loaded microgels were purified by a single cycle of centrifugation at 12,000 rpm and 10 °C for 1 h. The collected pink product was redispersed in deionized water under sonication for subsequent usage.

### 2.3. Synthesis of Ag@Au/PNIPAm/PEI Nanocomposite Particles

Synthesis of the Ag@Au bimetallic nanoparticles was carried out through a successive reduction of AgNO_3_ in the presence of the Au@PNIPAm/PEI particles. The preformed gold nanoparticles were used as seeds for the successive reduction of the silver ions to silver nanoparticles. A 1:1 molar ratio sample that contained 1 mL Au salt solution (1.317 × 10^−3^ M) and 1 mL Ag salt solution (1.317 × 10^−3^ M) was used in this study. The reaction was first carried out at room temperature for 30 min, followed by heating at 60 °C for another 30 min. During the mixing of the silver salt solution with the Au-loaded particles, the solution changed color from light pink to light gray, and eventually turned fully gray after heating the mixture.

### 2.4. Catalytic Activity of Nanocomposite Particles

The catalytic activity of both Au@PNIPAm/PEI and Ag@Au/PNIPAm/PEI nanocomposite particles were studied in an aqueous system using reduction of *p-*nitrophenol to *p-*aminophenol as a model reaction. Sodium borohydride (200 µL, 0.001 M), *p*-nitrophenol (30 µL, 0.001 M) and a definite quantity of the composite particles (40 µL, 1.5 wt % solid content) were diluted to 2 mL with deionized water and mixed in a cuvette reactor. The reaction mixture changed from light yellow to colorless. The catalytic reaction using Au@PNIPAm/PEI (amine to gold molar ratio of 28:1) and Ag@Au/PNIPAm/PEI [amine to gold and silver ions ratio (N/Au^3+^Ag^+^) of 28:1] were systematically studied under different solution pH values (3–11) and temperatures (25–39 °C). The reduction of *p-*nitrophenol to *p*-aminophenol was monitored by the UV-visible spectroscopy (Agilent Technologies, Santa Clara, CA, USA) at a wavelength of 400 nm for 20 min. Measurements were conducted at 2-min intervals.

### 2.5. Measurements and Characterization

#### 2.5.1. Particle Sizes and Surface Charges

The hydrodynamic diameter and *zeta*-potential of the microgel templates as well as mono- and bimetallic-loaded nanocomposite particles were all measured with a Beckman Coulter Delsa Nano particle analyzer (Beckman Coulter, Brea, CA, USA) using a photon correlation spectroscopy with electrophoretic dynamic light scattering (a two-laser diode light source with a wavelength of 658 nm at 30 mW). Hydrodynamic diameter, *D*_h_, was obtained from the Einstein Stokes equation, *D*_h_ = *kT*/3πη*D*, where *k* is the Boltzmann constant, η is the dispersant viscosity, *T* is the temperature (K), and *D* is the diffusion coefficient. The diffusion coefficient was obtained from the decay rate of the intensity correlation function of the scattered light (*i.e.*, correlogram), *G*(*τ*) = ∫*I*(*t*)*I*(*t* + *τ*)d*t*. Each measurement was carried out in triplicate. *zeta*-Potential measures the surface charge of particles based on their electrophoretic mobility. Samples for *zeta–*potential measurements were diluted to 100–200 ppm with 1 mM NaCl and measured at 25 °C.

#### 2.5.2. Transmission Electron Microscopy

Transmission electron microscopy (TEM) images of monometallic gold-loaded microgels and bimetallic (Ag@Au)-loaded microgels were observed using a transmission electron microscope (JEOL 100 CX, JEOL, Tokyo, Japan) at an accelerating voltage of 100 kV. The high resolution TEM micrographs of Au and Ag@Au nanoparticles and their corresponding selected area electron diffraction (SAED) patterns were characterized by a JEOL 2010 TEM (JEOL) at an accelerating voltage of 200 kV. The sample was prepared by wetting a carbon-coated grid with a 5 µL of the diluted particle dispersion, followed by drying at room temperature prior to TEM analysis. There was no pretreatment staining for all nanocomposite samples.

#### 2.5.3. X-ray Photoelectron Spectroscopy

X-ray photoelectron spectroscopy (XPS) data were recorded on a multi-surface analysis system (PHI 5600, Physical Electronics, Chanhassen, MN, USA) with a monochromatic AlK_α_ X-ray source (1486.6 eV). Sample spot sizes varied from 200 to 400 µm in diameter. The pass energies of exciting radiations were set at 187 and 45 eV for survey and elemental scans, respectively. The energy and emission currents of the electrons were 4 eV and 0.35 mA, respectively. Energy resolution was at 0.7 eV with a chamber pressure of 5 × 10^−10^ torr. Spectral calibration was determined by setting the C 1s component at 285.0 eV. All data acquisition was processed with a PC-based Advantage software (version 1.85, Advantage Software Co., Stuart, FL, USA). The surface composition was determined by using the manufacturer’s sensitivity factors. Curve fitting of the spectrum was accomplished using a nonlinear least-squares method. A Gaussian function was assumed for the curve fitting. The deconvolution of carbon, oxygen, and nitrogen peaks was processed with MagicPlot software (version 2.5.1, MagicPlot Systems, St. Petersburg, Russia).

#### 2.5.4. UV-vis Spectroscopy

UV-vis spectra were recorded on a Varian Cary 4000 Spectrophotometer using wavelengths ranging from 250–525 nm with an absorbance set from 0 to 1.40 a.u. Samples were diluted to appropriate concentrations and measured in the 5-mL cuvette. Actual absorbance as a function of time was plotted and a fitted curve was derived from an analysis-fitting function of Origin Pro software (v. 8.0, OriginLab Co., Northampton, MA, USA). Time-dependent UV-vis spectra (Agilent Technologies) on the reduction of *p*-nitrophenol to *p*-aminophenol using both monometallic (Au@PNIPAm/PEI) and bimetallic (Ag@Au/(PNIPAm/PEI)) nanocomposite particles as catalysts were used to monitor the catalytic reaction and calculate the catalytic constant, *k*. This constant is proportional to the catalytic rate of the reaction. UV-vis absorbance at 400 nm is a characteristic peak of the *p*-nitrophenol. During the course of the catalytic reaction, the intensity of this peak decreased due to its conversion to *p*-aminophenol, which appeared at wavelengths between 290 and 310 nm. The gradual reduction in UV-vis absorbance of the *p*-nitrophenol was monitored at specific time intervals until its full disappearance.

#### 2.5.5. Atomic Force Microscopy

Atomic force microscopy (AFM) images of PNIPAm/PEI microgel particles at 29 and 45 °C in aqueous solution were obtained using XE-120 inverted microscope complete AFM system with universal liquid cell option (Park Systems, Suwon, Korea). Imaging was carried out in a temperature control stage using a standard silicon nitride (Si_3_N_4_), gold-coated cantilever tip (MLCT-AUHW, Veeco, Plainview, NY, USA) in a non-contact fluid. The AFM system carries a decoupled XY-scanner with a maximum scan range of 100 μm × 100 μm. Image matrix was 256 × 256 pixels for fluid samples. Scan rate used was 0.5 to 0.8 Hz depending on the sample conditions. Humidity was adjusted from 40% to 80%.

#### 2.5.6. Elemental Analysis of Bimetallic Nanoparticles

The atomic percentage of individual bimetallic nanoparticle was evaluated using an Energy-dispersive Spectrometry (EDS) probe, operating in the bright field mode of a JEOL 2010 TEM. The spectral resolution was 1 nm. In the chemical composition measurement, Au showed energy intensities at 2 and 2.6 keV, whereas Ag intensities were at 3 and 3.4 keV.

## 3. Results and Discussion

### 3.1. Synthesis of Au and Ag@Au/PNIPAm/PEI Nanocomposite Particles

**Scheme 1:**
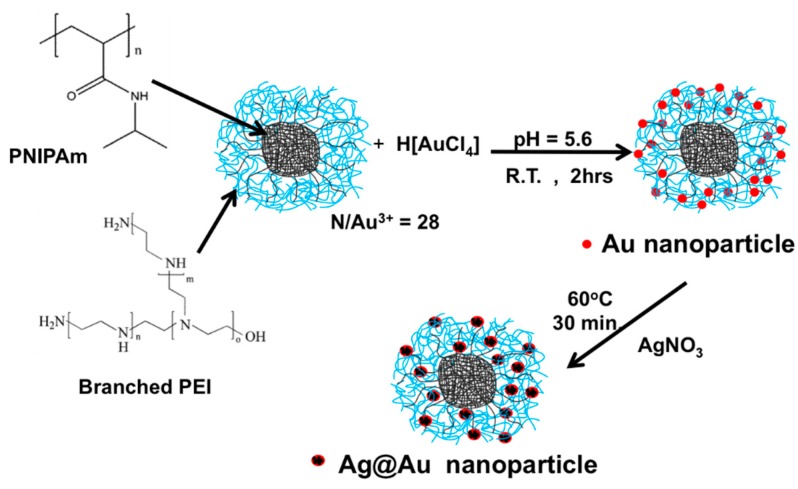
Reaction scheme of the formation of Au and Ag@Au metal nanoparticles using PNIPAm/PEI template.

The synthesis of both mono- and bimetallic nanoparticles is illustrated in Scheme 1. The gold nanoparticles were first generated through a reduction of gold salt with a polymeric amine using core-shell particles that consisted of poly(*N*-isopropyl acrylamide) cores and polyethyleneimine shells. Generation and stabilization mechanisms of gold nanoparticles and formation of Au@PNIPAm/PEI nanocomposite particles have been discussed in our previous papers [17,18]. Amine groups are known to have a reducing ability to generate metal nanoparticles [22]. They also can complex with metal ions and metal nanoparticles through their chelating properties [23,24]. When using the PNIPAm/PEI core-shell template, gold salt ions [AuCl_4_]^−^ were attracted into the template through an electrostatic interaction between the positively charged PEI shell and the negatively charged gold salt ions. The high local amine concentration could significantly enhance the reduction rate of gold salt ions to generate gold nanoparticles without the aid of any reducing agents.

Formation of the AuNPs was evident from the change in solution color from white to light pink, which occurred after 30 to 40 min of reaction at room temperature. These gold nanoparticles were then used as seeds for the successive reduction of silver ions to bimetallic Ag/Au nanoparticles using silver nitrate solution. The reduction was carried out at 60 °C in order to increase the conversion and crystallinity of the bimetallic nanoparticles. In the presence of gold metal nanoparticles, silver ions (Ag^+^) could be reduced to silver nanoparticles via an under-potential deposition mechanism [25], or, as others refer to it, the noble metal induced reduction (NMIR) method [26]. In this mechanism, the gold nanoparticles acted as seeds or active sites for further growth of silver nanoparticles. The formation of silver in gold bimetallic nanoparticles was evident upon a change in the solution color from light pink to gray. This effect is attributed to the fact that the ionization potential and electron affinity values of Au atom are higher than those of the Ag atom. The large electronegativity value of the Au atom leads to effective charge transfer from silver to gold atoms because the second metal ion (Ag^+^) has lower reduction potential than gold nanoparticles [27,28].

### 3.2. Compositions and Morphologies of Au and Ag@Au/PNIPAm/PEI Nanocomposite Particles

**Figure 1:**
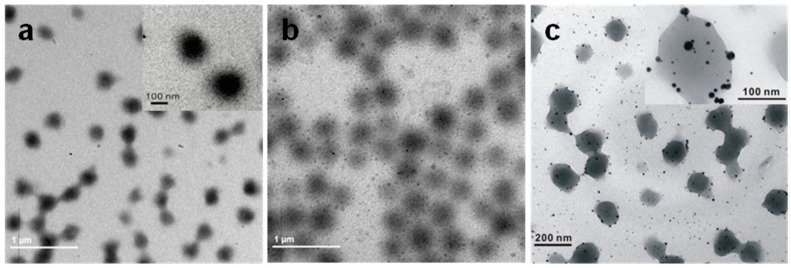
TEM images of (**a**) PNIPAm/PEI microgel templates; (**b**) Au/PNIPAm/PEI nanocomposite particles prepared at room temperature; (**c**) Ag@Au/PNIPAm/PEI nanocomposite particles formed at 60 °C (Au^3+^/Ag^+^ molar ratio of 1:1).

The TEM images shown in Figure 1 reveal the transformation of PNIPAm/PEI to Au and Ag@Au/PNIPAm/PEI nanocomposite particles. The PNIPAm/PEI particle displays a core-shell structure where the core has a darker contrast than the shell (Figure 1a inset). The resultant Au@PNIPAm/PEI particles are shown in Figure 1b. The gold nanoparticles look like clusters of small gold nanoparticles with an average diameter of 15 ± 4.0 nm. They were homogenously distributed within the particles. Figure 1c shows the morphology of Ag@AuPNIPAm/PEI nanocomposite particles. The bimetallic nanoparticles displayed two different intensities of contrast (Supplementary Materials Figure S1). The region in a darker shade of gray constitutes the gold nanoparticle while the lighter gray part is the silver nanoparticles. Elemental analysis results revealed that the bimetallic nanoparticles comprised 17.6 and 82.4 atomic percentage of Ag and Au, respectively (Supplementary Materials Figure S2). The higher Au percentage may be attributed to the fact that only partial Au^3+^ ions were initially reduced to gold nanoparticles at room temperature, while the remaining gold salt ions [AuCl_4_]^−^ were attracted to the positively charged PEI shells. When the temperature was raised to 60 °C, the other part of the gold salt ions was further reduced together with some Ag^+^ ions, resulting in the formation of Ag@Au nanoparticles with a higher Au content.

It was noted that the average size of the bimetallic nanoparticles was only *ca*. 6.83 ± 2.5 nm as shown in Figure 1c. Their sizes were much smaller than those of the original Au nanoclusters, indicating that the Au nanoclusters were dissociated after forming bimetallic nanoparticles. The smaller bimetallic nanoparticles were more difficult to entrap within the soft and flexible PEI shell. Thus some of them escaped from the templates and dispersed in the solution. Furthermore, the bimetallic nanoparticles located in the templates or freely dispersed in solution appeared much darker than the Au nanoclusters. This phenomenon may be attributed to the thermal treatment of the nanocomposite particles, a process that facilitates the growth of the metallic nanoparticles through further crystallization.

**Figure 2:**
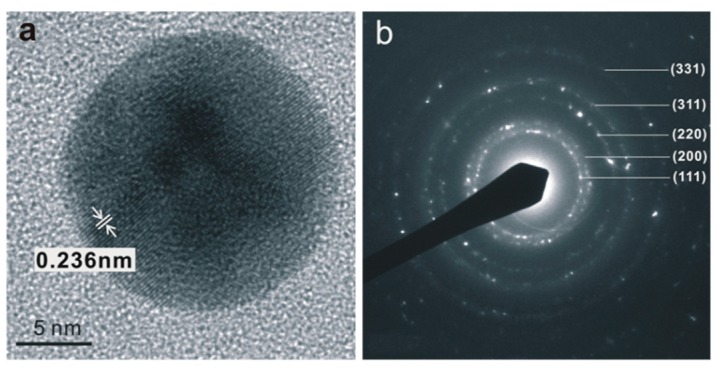

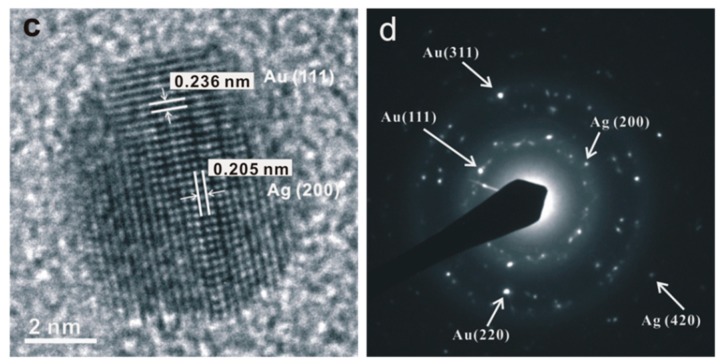
(**a**) HRTEM image of Au nanoparticle embedded within a microgel template with measured lattice *d*-spacing of 0.236 nm; (**b**) the selected area electron diffraction (SAED) pattern from several Au nanoparticles; (**c**) HRTEM image of a magnified single Ag@Au nanocrystal with corresponding lattice parameters of Au and Ag; (**d**) SAED of Ag@Au nanoparticles.

A high-resolution TEM (HRTEM) was performed to verify the nanostructures of mono- and bimetallic nanoparticles. In order to clearly observe the metal nanoparticles in the image, the nanocomposite particles were pre-treated with electron beam irradiation with a current density of 0.4 nA/nm^2^ for 30 s to partially remove the polymer template. Figure 2a shows an image of a polycrystalline Au nanostructure with a diameter of *ca*. 19.1 nm. Figure 2b shows the selected area electron diffraction (SAED) pattern over several Au nanoparticles. It reveals a ring pattern indexed as (111), (200), (220), (311), and (331) of a face-centered cubic (fcc) gold lattice. Thus, the gold nanoparticle is mainly composed of (111) planes with a *d*-spacing of 0.236 nm. The fuzzy central portion of the nanoparticle exhibited in the HRTEM image indicates that the Au nanoparticle adopts an icosahedral morphology with multiple-twinned structure [29]. Such defect is attributed to the small displacement of atoms located at the central nucleation site of the nanoparticle. Figure 2c,d show the crystallinity and different lattice arrangement of the Ag@Au bimetallic nanoparticles, respectively. The difference in electron densities between gold and silver is due to their differences in the corresponding lattice parameter. Au (111) has a lattice *d*-spacing of 0.236 nm, while that of Ag (200) is 0.205 nm. Hence, the SAED analysis of the bimetallic nanoparticles further confirmed the co-existence of the crystalline orientations of Au and Ag in a single nanoparticle.

### 3.3. Particle Sizes and Surface Charges of the Nanocomposite Particles

Changes in particle sizes and surface charges of the original PNIPAm/PEI template, the Au@PNIPAm/PEI, and the Ag@Au/PNIPAm/PEI nanocomposite particles have been examined by measuring their hydrodynamic sizes and *zeta-*potential values. The results in Table 1 show that the introduction of gold ions into the PNIPAm/PEI template decreases both the particle size and the surface charge. The addition of Ag^+^ ions to form bimetallic nanocomposite particles further decreases both the particle size and the surface charge. These effects may be attributed to the strong binding affinity of the Au or Ag@Au nanoparticles with the amine groups, resulting in the contraction of a highly swollen PEI shell. The conversion of hydrophilic amino groups to more hydrophobic moieties or crosslinking of the PEI chains due to the formation of amine radical cation during the gold ion reduction is another possible reason [22]. Despite the decrease of surface charges to almost +5 mV, no precipitation was observed during the synthesis. However, slight aggregation of the nanocomposite particles was observed after several days of the reaction. These aggregates could be easily re-dispersed back to a stable colloidal system after sonication. Thus, the PEI and PNIPAm graft chains located in the particle shell were able to provide both electrostatic and steric stabilizations of the resultant nanocomposite particles.

### 3.4. Surface Chemical Composition of Nanocomposite Particles

**Figure 3:**
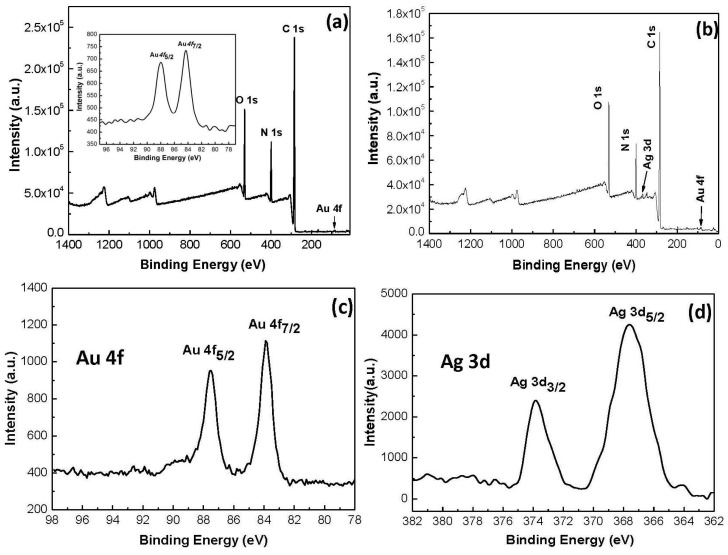
(**a**) Survey scan of Au/PNIPAm/PEI microgel. Inset is Au *4f* XPS spectrum of Au nanoparticles; (**b**) Survey scan of Ag@AuPNIPAm/PEI microgel film (Au/Ag of 50/50 mol. ratio); (**c**) Au *4f* XPS spectrum; and (**d**) Ag *3d* XPS spectrum of Ag@Au bimetallic nanoparticles.

Surface chemical compositions of both the Au@PNIPAm/PEI and the Ag@Au/PNIPAm/PEI nanocomposite particles were characterized by XPS analysis. This technique detects elements at a depth of 10 nm, and provides surface chemical information based on photoemission of electrons induced by X-rays. Figure 3a shows the XPS spectrum of the Au/PNIPAm/PEI nanocomposite particles. The survey spectrum reveals the characteristic binding energy peaks of C, O, N, and Au. Deconvoluted C 1s, O 1s, and N 1s peaks are shown in Figure S3 in the Supplementary Materials. The deconvoluted C 1s peaks at 285.0 and 287.7 eV are assigned to C–C/C–H and C=O bonds. The deconvoluted O1s peaks at 531.6 and 533.3 eV are assigned to the amide as well as hydroxyl functional groups which come from physically absorbed H_2_O. The deconvoluted N 1s peaks at 399.6 and 401.1 eV are assigned to the nitrogen from amine and amide. The XPS elemental peak profile (Figure 3a, inset) of gold nanoparticles is confirmed based on two characteristic peaks at 84.3 and 88.0 eV, which correspond to the Au *4f_7_*_/2_ and Au 4*f*_5/2_ of elemental gold at zero oxidation state. These results suggest that the particle shell contains components of PEI, PNIPAM and gold nanoparticles.

Figure 3b shows the XPS spectrum of the Ag@Au/PNIPAm/PEI nanocomposite particles. The survey spectrum reveals the characteristic binding energy peaks of C, O, N, Au, and Ag. Deconvoluted C 1s, O 1s and N 1s peaks are shown in Figure S4 in the Supplementary Materials. Figure 3c shows two peaks at binding energies of about 83.8 and 87.5 eV, which were assigned to Au 4*f*_7/2_ and Au 4*f*_5/2_ of zero-valent gold (Au^0^), respectively. Figure 3d shows the two peaks at binding energies of 367.6 and 373.8 eV, which corresponded to Ag 3*d*_5/2_ and Ag 3*d*_3/2_ of metallic Ag^0^, respectively. The differences between the *4f*_7/2_ and *4f*_5/2_ peaks for gold nanocrystal (3.6 eV) and between the *3d*_5/2_ and *3d*_3/2_ peaks for silver nanocrystal (~6.0 eV) were of similar values of zero valent gold and silver as reported in the literature [30]. These results, in agreement with the results of the HRTEM analysis, suggest that both Au and Ag components were located in the shell region.

### 3.5. Catalytic Properties of Au and Ag@Au PNIPAm/PEI Nanocomposite Particles

The catalytic activities of the nanocomposite particles were studied through a model reaction to reduce *p*-nitrophenol to *p*-aminophenol with sodium borohydride. The reaction was monitored by a UV-vis spectrometer since *p-*nitrophenol has a characteristic maximum absorption at 400 nm. A pseudo-first order kinetic reaction was chosen to derive the rate of catalytic reaction. At an excess amount of the reducing agent, NaBH_4_ (mole ratio of NaBH_4_:*p*-nitrophenol = 6.7:1), the rate of reaction constant, *k* of a pseudo-first order kinetic model can be described by Equation (1):

(1) $$\frac{-\;d_{c}}{d_{t}}=kC_{t}$$

where the absorbance of *p*-nitrophenol at *t* = 0 (*A*_0_) and at t (*A*_t_) are proportional to its initial concentration (*C*_0_) and concentration at time t (*C*_t_), respectively. Plotting *ln (C*_t_/*C*_o_*) versus* time will give the rate constant based on the slope of *k (s*^−1^*)*.

In a control experiment, the *p*-nitrophenol reacted only with sodium borohydride to form *p*-aminophenol as the sole product. Changes in peak intensity at 400 nm were monitored in intervals of every two minutes (Supplementary Materials, Figure S5). It was found that there was little change in peak intensity after up to 20 min of reaction, indicating a very slow reduction reaction. The rate constant derived from the slope of the linear curve was 5.4 × 10^−3^·s^−1^.

**Figure 4:**
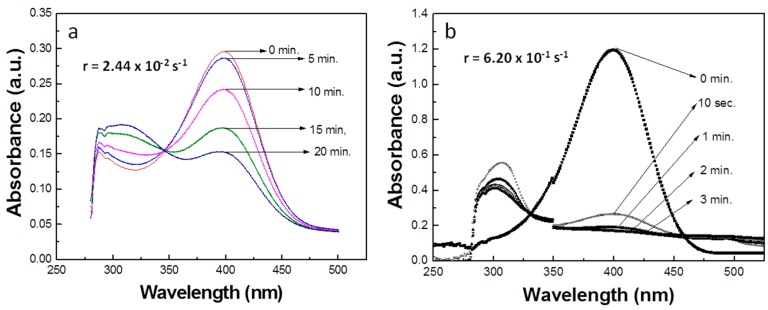
(**a**) Time-dependent UV-vis spectra on the reduction of *p-*nitrophenol to *p*-aminophenol. (**a**) Use of Au@PNIPAm/PEI nanocomposite particles as catalyst; (**b**) use of Ag@Au/PNIPAm/PEI nanocomposite particles as catalyst.

**Figure 5:**
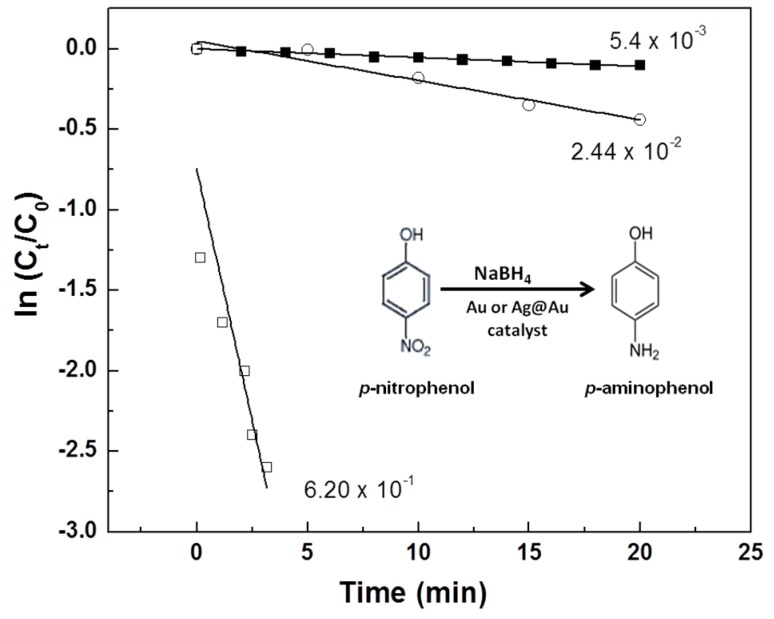
Plots of ln (*C*_t_/*C*_0_) as a function of time for the reaction at room temperature: (■) Without catalyst; (○) In the presence of Au@PNIPAm/PEI (N/Au molar ratio = 28); (□) In the presence of Ag@Au PNIPAm/PEI (N/AgAu molar ratio = 28).

When Au@PNIPAm/PEI nanocomposite particles were used, the reaction proceeded approximately 4.5 times faster (2.44 × 10^−2^·s^−1^) than without the immobilized AuNPs (Figure 4a). When Ag@Au/PNIPAm/PEI nanocomposite particles were used in the same reaction system, the reaction rate was significantly enhanced, as shown in Figure 4b. The reduction rate constant was 6.20 × 10^−1^·s^−1^, indicating a much faster reaction rate than the monometallic gold nanoparticle system. Figure 5 shows the corresponding catalytic reaction rates by plotting ln(*C*_t_/*C*_0_) *versus* reaction time for the reduction of *p-*nitrophenol to *p*-aminophenol. These results indicate that the bimetallic nanocomposite particles gave a reaction 25 times faster than using monometallic gold nanocomposite particles and more than 100 times faster than the reaction without nanocomposite particles. In fact, the catalytic performance of the Ag@Au/PNIPAm/PEI nanocomposite particles was superior to other supported Ag@Au bimetallic nanoparticles reported in the literature. For examples, catalytic rate constants of a metal–organic framework-supported Au@Ag ([31], polystyrene-supported Ag@Au [32], and graphene oxide supported Au-Ag alloy [33] were 4.97 × 10^−3^·s^−1^, 15.47 × 10^−3^·s^−1^ and 0.05 s^−1^, respectively. The substantial enhancement in catalytic activity may be attributed to the synergistic effect of the bimetallic nanoparticles derived from their unique electronic and geometrical properties [31,34,35]. It was also suggested that the increase in the number of low coordination number edge site of Ag and corner sites of Au could enhance the catalytic activity [36].

### 3.6. Stimuli-Responsive Properties of PNIPAm/PEI Template and Tuneable Catalytic Activities of the Nanocomposite Particles

**Figure 6:**
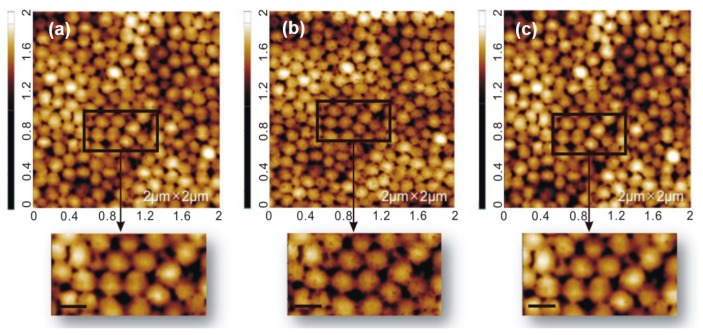
AFM micrographs of PNIPAM/PEI microgel particles measured in a fluid mode at different temperatures: (**a**) 29 °C; (**b**) 45 °C; and (**c**) cooled from 45 to 29 °C. (Scale bar: 200 nm).

One of the unique properties of the PNIPAm/PEI is its ability to respond to the dual stimuli of pH and temperature. As discussed earlier with reference to the XPS results, there were some PNIPAm chains that co-existed with PEI chains in the particle shell. To verify the morphological changes of the microgels both below and above the volume phase transition temperature (VPTT) of 32 °C, we used atomic force microscopy (AFM) in a fluid mode to observe their morphologies in an aqueous solution. Figure 6a shows an image of the microgel particles at 29 °C with particle sizes in the range of 150 to 200 nm and smooth surface morphology. When the temperature was raised to 45 °C, which is above the VPTT of the microgels, they became not only smaller (100–150 nm), but also had a porous surface form (Figure 6b). The particle size reduction was attributed to the shrinkage of the microgels above their VPTT. The porous surface was generated due to the contraction of the PNIPAm chains located in the shell. When the temperature was cooled down back to 29 °C, the smooth morphology and the original size of the microgel particles were restored. These results suggest that manipulation of solution temperature could induce conformational changes of the microgel with good reversibility. Thus we envisaged that the shrinking and expanding action of the nanocomposite particles could be used to control the catalytic activity of the metallic nanoparticles immobilized within the PNIPAm/PEI microgel through limiting and maximizing the exposure of the metal nanoparticles surface to reactants [37].

**Figure 7:**
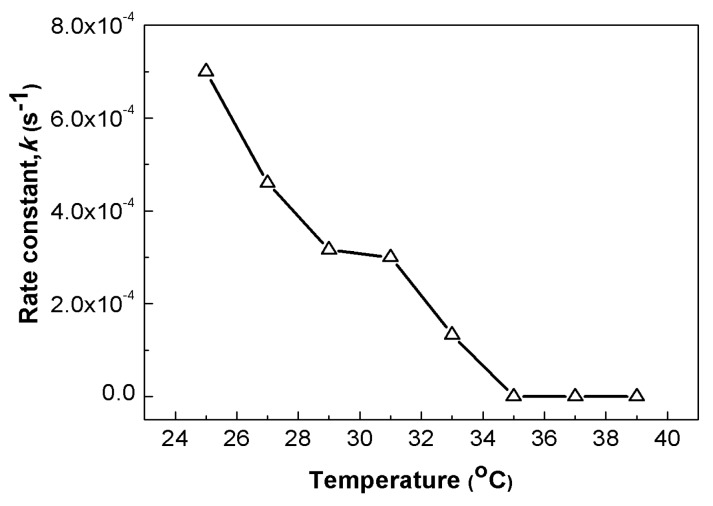
Effect of temperature on the rate constant of Au@PNIPAm/PEI nanocomposite particles for catalytic reduction of *p-*nitrophenol to *p-*aminophenol at pH = 5.6.

The effect of temperature on the catalytic activity of the Au@PNIPAm/PEI nanocomposite particles is shown in Figure 7. Catalytic activity is at the highest value at 25 °C, and decreases as temperature increases, eventually ceasing when the temperature is above 35 °C. The decrease in catalytic activity can be explained based on the accessibility of reactants to gold nanoparticles at different temperatures. When the solution temperature is below the VPTT of the Au@PNIPAm/PEI nanocomposite particles, the template swells and the gold nanoparticles become more accessible under this condition. However, when the temperature is above the VPTT of the nanocomposite particles, the PNIPAm core shrinks notably, resulting in the “dragging in” of the whole microgel particle and the covering of the catalyst surface. As a result, the reactant molecules are more difficult to penetrate and interact with the surface of gold nanocatalyst embedded in the nanocomposite particles for the reaction to proceed. The catalytic activity eventually ceased when most of the surface of the gold nanoparticles was covered by the polymer.

**Figure 8:**
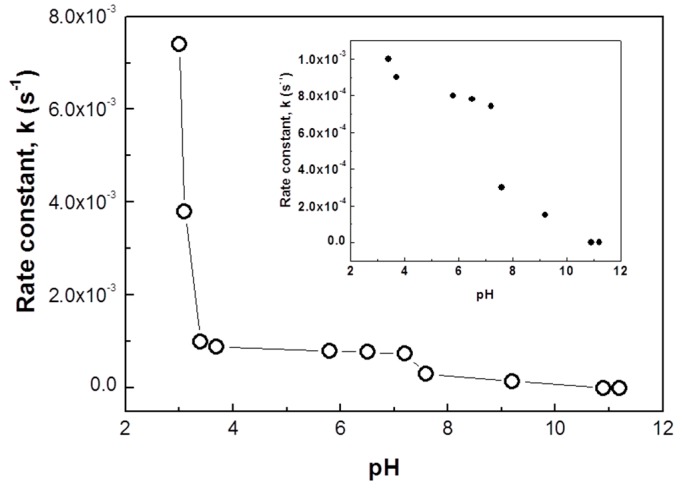
Effect of pH on the rate constant of Au@PNIPAm/PEI nanocomposite particles for catalytic reduction of *p-*nitrophenol to *p-*aminophenol at room temperature.

The effect of solution pH on the catalytic activity of the Au@PNIPAm/PEI nanocomposite particles was also systematically studied from pH 3 to 11. Results shown in Figure 8 indicate that the highest catalytic activity is at around pH 3 with a corresponding rate constant of 7.5 × 10^−3^·s^−1^. This activity is almost 10 times higher than at the neutral pH (7.4 × 10^−4^·s^−1^). An abrupt reduction in catalytic activity was found in the pH range of 3.0–3.5. Further increasing the solution pH to 7.5 has little influence on the catalytic activity. When increasing the pH to 11, its catalytic activity ceases. To ensure that the acid-catalyzed reaction was minimal or had no effect at all, a control experiment was performed at pH 3 in the absence of nanocomposite particles. The reduction rate of *p-*nitrophenol to *p-*aminophenol at pH 3 was found to be 1.52 × 10^−5^·s^−1^. This value is much smaller than the reaction using Au/PNIPAm/PEI nanocomposite particles (reaction rate was 7.5 × 10^−3^·s^−1^) at the same pH of 3. The results confirmed that an enhanced reaction rate at pH 3 was not caused by the acid-catalyzed reaction.

The variation of catalytic activity may be attributed to the pH-responsiveness of the PEI shell of the nanocomposite template. It is known that the percentage of protonated amines varies with the solution pH [38]. For example, percentages of protonated amines at pH 3, 7, and 9 are around 75%, 25%, and 8%, respectively. Therefore, the effect of solution pH on the catalytic activity of the Au@PNIPAm/PEI nanocomposite particles may be explained by the following reasons: Under low acidic pHs (pH = 3–4), a high protonation degree of the amino groups occurs, resulting in the increase of charge density and the stretching of the PEI network. The expanded PEI shell provides more exposure for the gold nanoparticle to interact with reactant molecules. On the other hand, increasing the solution pH leads to lowering the protonation degree, thus forming a more compacted PEI shell. Consequently, the shielding of the PEI shell makes it difficult for the reacting species to diffuse into the catalytic surface of the gold nanoparticles. These results demonstrate that the catalytic activity of Au@PNIPAm/PEI nanocomposite particles can be easily turned “on” and “off” by adjusting the solution pH.

### 3.7. Reusability of Nanocomposite Particles

**Figure 9:**
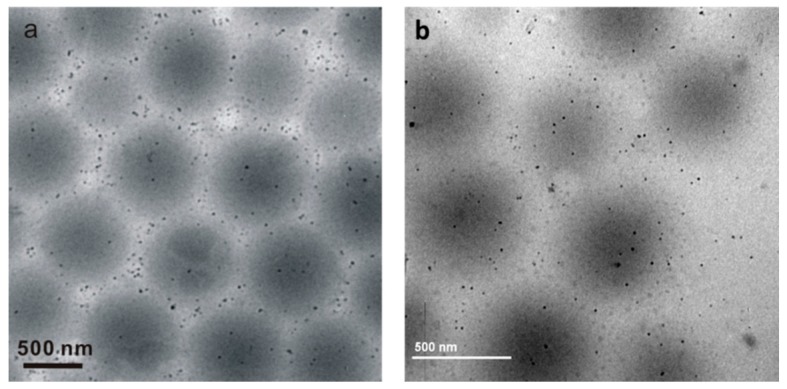
TEM images (**a**) before catalysis and (**b**) after catalysis. The Au/PNIPAm/PEI nanocomposites was recovered at 10,000 rpm centrifugation for 1 hour, followed by redispersing the particles in deionized water.

The reusability of Au/PNIPAm/PEI nanocomposite particles has been examined by comparing morphologies of nanocomposite particles before and after the catalytic reactions. The Au/PNIPAm/PEI nanocomposite particles were recovered after one cycle of catalytic reaction through centrifugation and redispersion. TEM images shown in Figure 9 reveal that the morphology of the recovered nanocomposite particles is quite similar to those original nanocomposite particles. These results suggest that the nanocomposite particles may be reusable for catalytic reaction.

## 4. Conclusions

We have developed a simple route for *in situ* synthesis of Au and Ag@Au nanoparticles using smart PNIPAm/PEI core-shell microgel particles as dual reductant and template. The gold nanoparticles were initially formed through a reduction of gold ions with highly concentrated amine functional groups of the microgel. The resultant gold nanoparticles were then used as seeded nanoparticles for further reduction of silver ions to form bimetallic alloy nanoparticles. The use of polymeric amine-based particles to produce metal/polymer nanocomposite particles in an aqueous solution is a simple and green synthesis without using any organic solvent, reducing or stabilizing agents. The resulting nanocomposite particles possess not only excellent catalytic activities with good reproducibility and stability, but also smart properties that allow the tuning of catalytic activities of the metal nanoparticles through varying solution pH and temperature. Therefore, these metal nanoparticle immobilized nanocomposite particles possess high potential for practical applications in catalysis.

## Figures and Tables

**Table 1:** Particle size and *zeta*-potential of different nanocomposite particles.

Type	Mean hydrodynamic diameter	*zeta*-Potential
	(nm)	(mV)
PNIPAm/PEI	384	+34
Au@PNIPAm/PEI	284	+15
Ag@Au/PNIPAm/PEI	270	+5

## Supplementary Materials

The supplementary materials can be found at www.mdpi.com/2073-4360/8/4/105/s1.
