# Supplementary material for: Green Synthesis of Smart Metal/Polymer Nanocomposite Particles and Their Tuneable Catalytic Activities
Source: Polymers (Basel). 2016 Mar 23;8(4):105. doi: 10.3390/polym8040105 (PMC6432224; doi:10.3390/polym8040105)
Supplement: Supplementary file 1 [file polymers-08-00105-s001.pdf]

Supplementary Materials: Green Synthesis of Smart Metal/Polymer Nanocomposite Particles and Their Tuneable Catalytic Activities

Noel Peter Bengzon Tan, Cheng Hao Lee and Pei Li

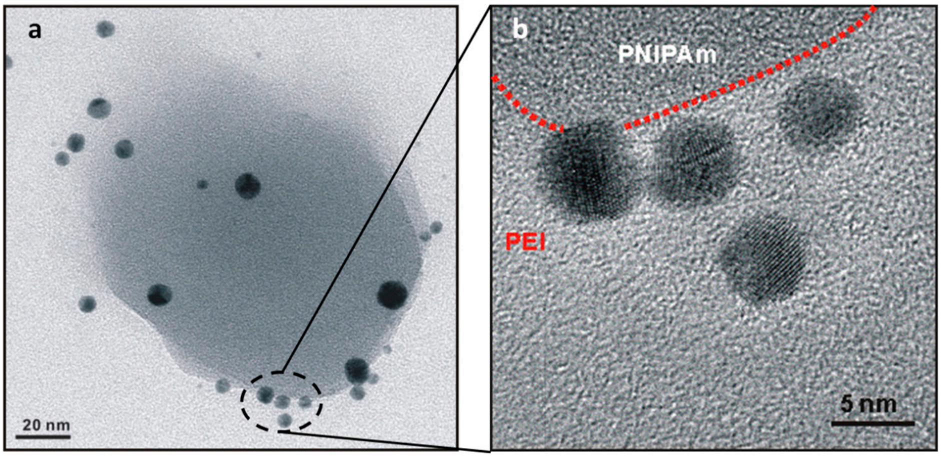

Figure S1. (**a**) TEM images of (**a**) Single PNIPAm/PEI particle template containing Ag@Au bimetallic nanoparticles; (**b**) The Ag@Au bimetallic nanoparticles observed under higher magnification.

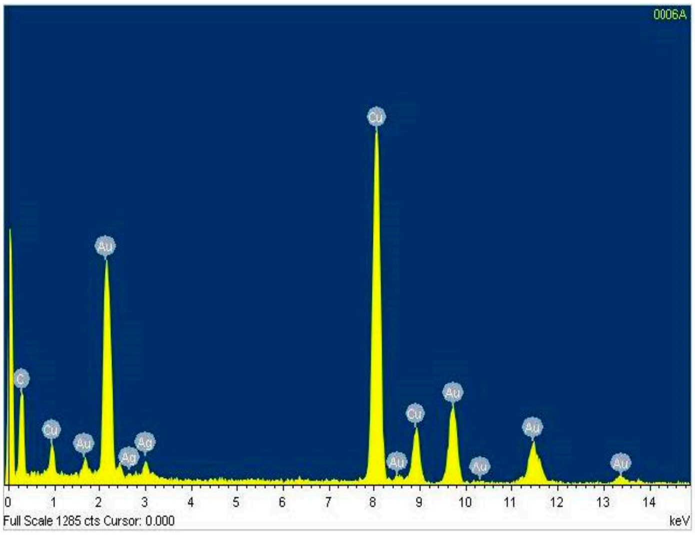

Element	Weight %	Atomic %
C K	13.25	69.75
Ag L	9.09	5.33
Au M	77.66	24.93

Figure S2. Elemental analysis of bimetallic nanoparticles indicates that the Ag and Au atomic% was 17.6 and 82.4, respectively.

1. Elemental XPS Spectra of Au and Au@Ag in PNIPAm/PEI

Elemental XPS peaks are shown in Figures S3 and S4 of both Au and Ag@Au in microgel template. C 1s peaks at 285.0 and 287.7 eV are assigned to C–C/C–H and C=O bonds. N1s peaks at 399.6 and 401.1 eV are assigned to the nitrogen from amine and amide. O 1s peaks at 531.6 and 533.3 eV are assigned to the amide and hydroxyl (O–H) functional groups.

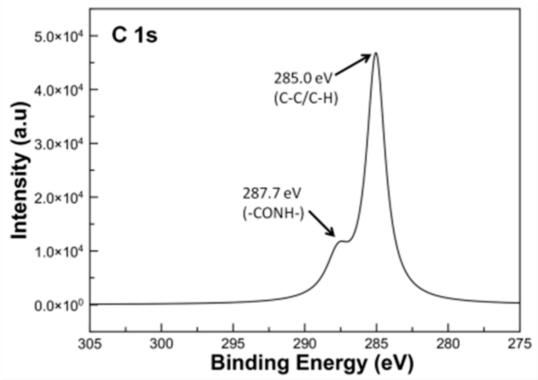

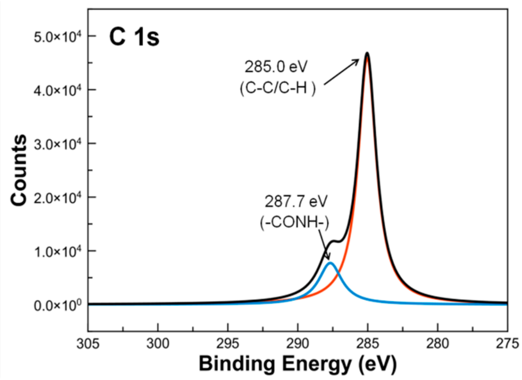

(**a**) (**b**)

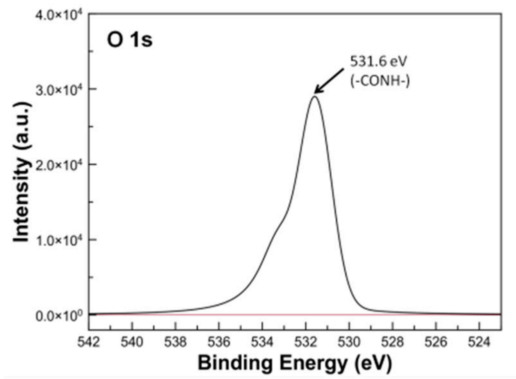

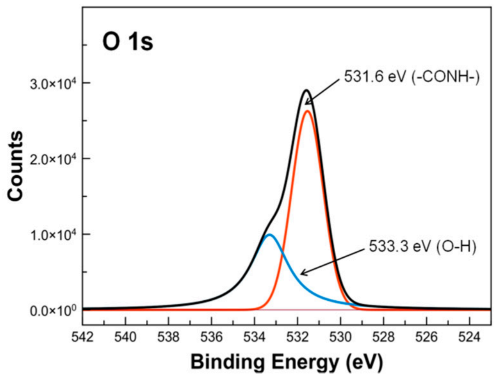

(**c**) (**d**)

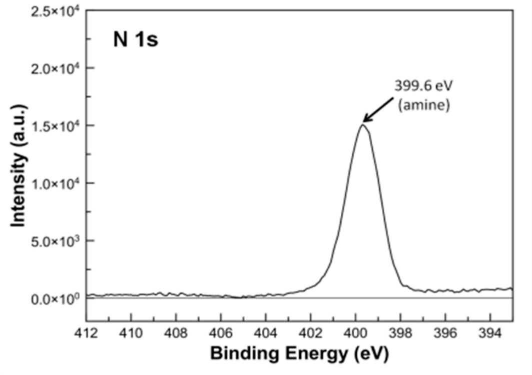

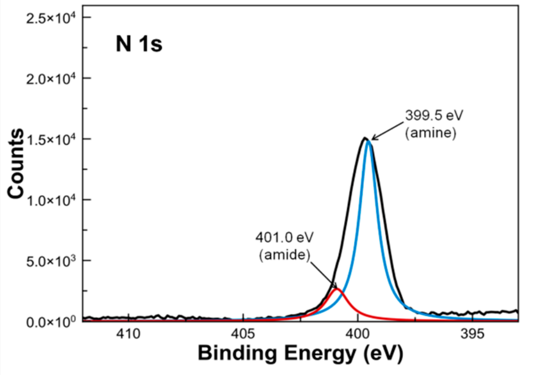

(**e**) (**f**)

Figure S3. XPS spectra of C 1s (**a**,**b**), O 1s (**c**,**d**) and N 1s (**e**,**f**) peaks and corresponding deconvoluted peaks of the Au@PNIPAm/PEI.

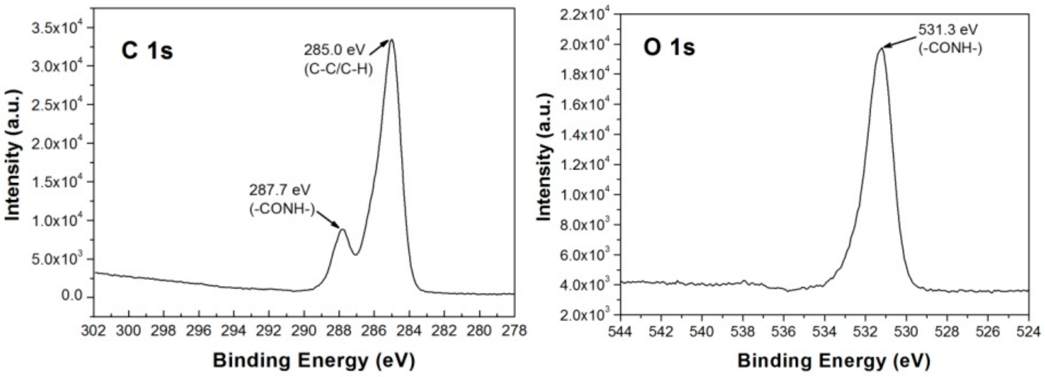

Figure S4. *Cont.*

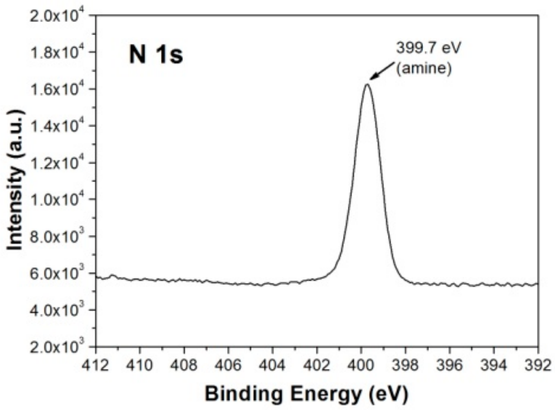

Figure S4. Deconvoluted XPS spectra of C 1s, N 1s and O 1s peaks of the Ag@Au/PNIPAm/PEI.

2. UV-vis Spectroscopy and Plot of ln (C_t/C_0) as a Function of Time of Control Experiment for the Reduction of *p*-Nitrophenol to *p*-Aminophenol

The reaction model, reduction of *p*-nitrophenol to *p*-aminophenol with sodium borohydride solution was used to demonstrate the catalytic reaction of the bimetallic nanoparticles. A control experiment was conducted by not using any catalyst for the said reaction. This was performed to compare the rate of reactions using the mono and bimetallic nanoparticles. Using a first order reaction, the slope of plotting ln (C_t/C_0) with time gives the constant for the rate of reaction. Without the use of any catalyst, the constant for the rate of reaction was determined as 5.4×10^{-3}.

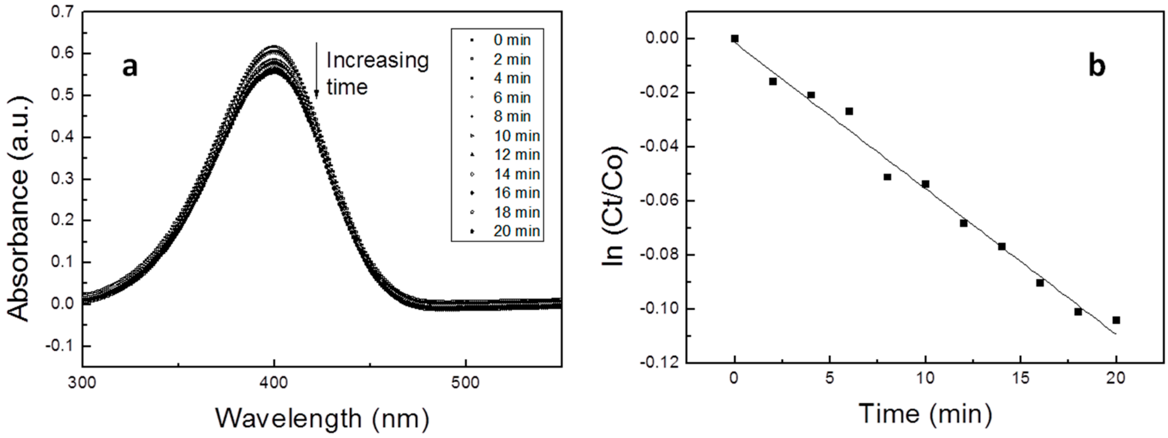

Figure S5. (**a**) UV-vis spectra of the sodium borohydride reduction of *p*-nitrophenol to *p*-aminophenol in the absence of a catalyst; (**b**) Plot of ln (C_t/C_0) as a function of time of control experiment for the reduction of *p*-nitrophenol to *p*-aminophenol.

© 2016 by the authors; licensee MDPI, Basel, Switzerland. This article is an open access article distributed under the terms and conditions of the Creative Commons by Attribution (CC-BY) license (http://creativecommons.org/licenses/by/4.0/).
